# Post‐Microbial Therapeutics for the Next Generation of Medicine

**DOI:** 10.1002/mbo3.70427

**Published:** 2026-09-30

**Authors:** Mohammad Nazrul Islam Bhuiyan, Maliha Momtaj

**Affiliations:** ^1^ BCSIR Chattogram Laboratories BCSIR Chattogram Bangladesh; ^2^ Bangladesh Council of Scientific and Industrial Research (BCSIR) Institute of Food Science and Technology Dhaka Bangladesh; ^3^ Bangladesh Council of Scientific and Industrial Research (BCSIR) Biomedical and Toxicological Research Institute (BTRI) Dhaka Bangladesh

**Keywords:** bacteriophage engineering, biofilm disruption, ecological dose, metabolic reprogramming, microbiome metabolites, post‐microbial therapeutics, precision microbiome medicine

## Abstract

Post‐microbial therapeutics is proposed here as an integrative, function‐centered framework rather than a new therapeutic class. It asks whether a defined microbial perturbation can produce a reproducible change in a microbial function, biochemical output, and clinically relevant host phenotype. Bacteriophages provide a perturbation model because their host dependence permits strain‐selective intervention, but the framework also distinguishes between whole phages and engineered phages, lysins, depolymerases, delivery systems, and combination products. A central requirement is that a microbial module must be defined by experimentally testable functional contribution rather than by statistical co‐occurrence alone. Computationally inferred modules are therefore treated as hypotheses until supported by perturbation, reconstruction, removal/add‐back, flux, or rescue experiments. Evidence reviewed across infection, gastrointestinal, biofilm, and cardiometabolic contexts shows a recurring pattern: phage exposure can alter bacterial abundance, resistance phenotypes, community interactions, and metabolism, but the strength of evidence diminishes as claims shift from bacterial killing to ecosystem restoration and host benefit. Engineering and delivery technologies can improve targeting or exposure, yet they introduce additional constraints in genetic stability, resistance, formulation, pharmacology, and regulation. The framework is consequently best viewed as a testable translational architecture that connects intervention to function and outcome while separating established evidence from emerging hypotheses.

## Graphical abstract

Post‐microbial therapeutics: from microbial perturbation to host benefit. Post‐microbial therapeutics provide a function‐centered framework that links targeted microbial perturbation to changes in microbial modules and metabolites, followed by measurable host responses. Phages and phage‐derived interventions can selectively modify bacterial populations and microbial functions, potentially reshaping metabolite profiles and host physiology. The framework emphasizes causal validation, functional outcomes, and iterative optimization rather than assuming that changes in microbial abundance or metabolites alone confer therapeutic benefit.

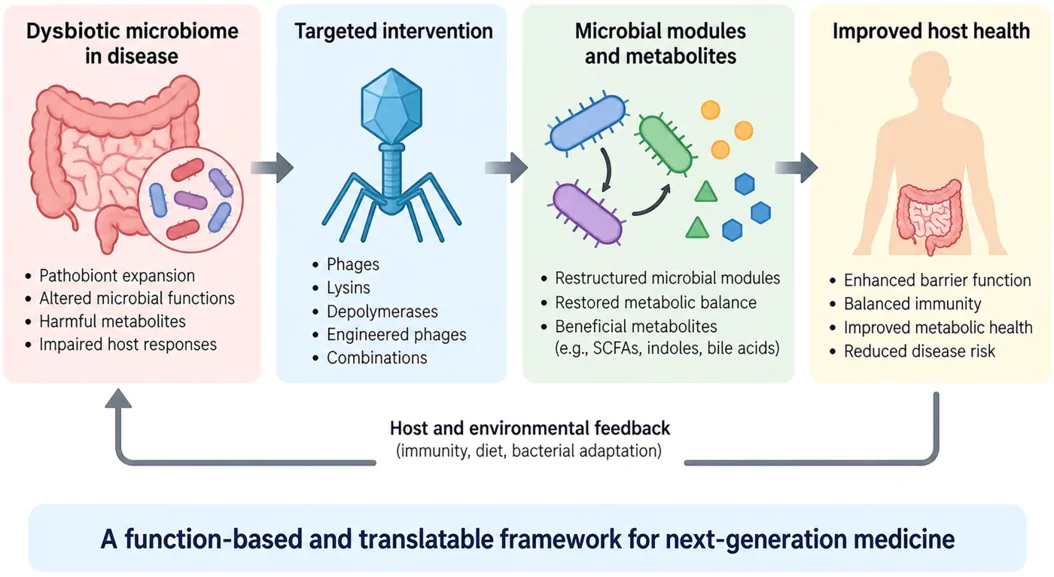

## 1 Introduction

Antimicrobial therapy has traditionally followed a pathogen‐centered objective: identify and eliminate the causative microorganism. Although antibiotics remain indispensable, their extensive use can promote antimicrobial resistance (AMR), microbiome disruption, recurrent dysbiosis, and treatment failure in biofilm‐associated or multidrug‐resistant infections (Pires et al. 2020; Strathdee et al. 2023). These limitations highlight the need for therapeutic strategies that consider not only pathogen abundance but also microbial interactions, metabolic activity, spatial organization, and host responses.

Systems biology and multiomics have consequently shifted microbiome research from taxonomy toward function. Host‐associated microbial communities form interconnected systems involving microorganisms, bacteriophages, metabolites, host immunity, and environmental substrates. Phage–bacterium interactions can alter bacterial physiology and community structure, whereas microbiota‐derived metabolites influence epithelial, immune, and metabolic processes (Federici et al. 2021; Hendrix et al. 2022; Malogan et al. 2025). These observations support a function‐oriented therapeutic perspective in which microbial activity and its consequences are considered alongside microbial identity.

Here, post‐microbial therapeutics are proposed as an integrative and operational framework rather than as a wholly new therapeutic class. It differs from the broader field of microbiome therapeutics, which includes interventions that modify microbiome composition or activity, and from ecological medicine, which considers host‐associated ecosystems more broadly. It also extends function‐based microbiome interventions by imposing a defined translational sequence linking intervention → microbial function → biochemical consequence → host outcome. The framework, therefore, does not claim novelty for individual modalities; its proposed contribution is to provide a common experimental architecture for selecting interventions, defining functional targets, measuring intermediate responses, and testing causal relevance.

The framework centers on three linked elements: the perturbation, the functional microbial unit, and the measurable biochemical or host response. Phages provide a useful model perturbation because their bacterial targets can sometimes be experimentally defined, although lysins, depolymerases, engineered phages, phage cocktails, or combination therapies may be preferable in particular settings. The framework is therefore intervention‐agnostic rather than inherently phage‐specific.

A microbial module is defined here as a functionally bounded microbial unit that contributes demonstrably to a specified biological process and produces a measurable output. Metabolic guilds, cross‐feeding consortia, and network clusters are not considered equivalent by default. Co‐occurrence, correlation, or network structure may identify candidate members, but module membership requires evidence of functional contribution or dependency. Candidate modules inferred computationally from multiomics or network data should therefore be distinguished from experimentally validated modules supported by perturbation, reconstruction, add‐back, isotope tracing, pathway‐specific flux analysis, metabolite rescue, or comparable causal evidence.

The resulting therapeutic logic is as follows: clinical or functional abnormality → candidate microbial function → candidate module → experimental validation → intervention → perturbation → functional/metabolic measurement → host‐response validation → clinical outcome.

This sequence distinguishes mechanistic intervention from simple taxonomic or metabolomic association. A change in microbial abundance, network structure, or metabolite concentration alone does not establish that a therapeutically relevant ecological transition has occurred.

Phages illustrate this distinction particularly well. Their activity depends on receptor availability, bacterial physiology, environmental conditions, antiviral defense systems, and evolutionary adaptation (Gordillo Altamirano and Barr 2021; Venturini et al. 2022). Phage specificity should therefore be considered at both the microbiological level, which strains can be infected, and the functional level, which community functions are altered. Virulent and temperate phages also require different therapeutic considerations because lysogeny can alter bacterial phenotype, fitness, and gene exchange. Genome screening and phenotypic characterization are consequently integral to therapeutic definition and safety assessment (Pires et al. 2020; Strathdee et al. 2023; Venturini et al. 2022).

Phage‐derived products should likewise be distinguished mechanistically. Whole phages are replicating biological agents; lysins are nonreplicating peptidoglycan‐hydrolyzing enzymes; depolymerases primarily target extracellular polysaccharides or capsule structures; and engineered phages introduce additional genomic and payload‐related variables. These differences affect pharmacology, manufacturing, evidence requirements, and regulatory considerations.

Evidence supporting the proposed framework remains uneven. Phage–antibiotic studies demonstrate metabolic reorganization, while experimental targeting of high‐alcohol‐producing *Klebsiella pneumoniae* has linked selective phage intervention with changes in a disease‐associated phenotype (Gan et al. 2023; Han et al. 2022; Hendrix et al. 2022). However, reproducible evidence that phage‐induced changes in defined microbial modules or metabolites mediate clinical benefit in humans remains limited. Accordingly, this review distinguishes direct antibacterial evidence, mechanistic preclinical evidence, associative ecosystem evidence, and prospective engineering concepts.

Finally, clinical translation remains constrained by phage pharmacokinetics/pharmacodynamics (PK/PD), immune clearance, tissue distribution, bacterial susceptibility, biofilm physiology, and resistance evolution. The concept of an ecological dose is therefore presented as a research hypothesis rather than a validated clinical dosing metric or replacement for conventional PK/PD. Together, these considerations position post‐microbial therapeutics as a testable framework for linking microbial perturbation to function and host outcomes while maintaining appropriate boundaries between established evidence and future therapeutic possibilities.

## 2 Phage Therapy: Historical Context, Modern Perspectives, and Future Trajectories

Bacteriophage therapy predates the antibiotic era and represents one of the earliest forms of targeted biological control of bacterial infection. Its use declined in much of Western medicine after the widespread adoption of antibiotics, but it persisted in select clinical settings and has regained attention amid the escalation of AMR. Contemporary phage therapy differs substantially from its early empirical use. Genome sequencing, receptor characterization, quantitative pharmacology, synthetic biology, and improved manufacturing now permit more systematic assessment of phage identity, host range, activity, safety, and therapeutic suitability (Pires et al. 2020; Strathdee et al. 2023). Phage therapy is therefore increasingly viewed as a precision biological intervention whose performance depends on interactions among phage biology, bacterial physiology, host immunity, pharmacology, microbial ecology, and evolutionary dynamics.

### 2.1 Resurgence in the Era of AMR

The global AMR crisis has renewed interest in bacteriophages as complementary or alternative agents for difficult‐to‐treat bacterial infections. Clinical studies, compassionate‐use experience, and systematic reviews have reported encouraging safety and case‐specific therapeutic benefit, particularly for infections caused by multidrug‐resistant organisms (Petrovic Fabijan et al. 2023; Stacey et al. 2022; Strathdee et al. 2023). However, the evidence remains heterogeneous across bacterial species, patient populations, phage preparations, routes of administration, concomitant antibiotics, and clinical endpoints. Phage therapy should therefore be considered a promising but still incompletely standardized therapeutic modality rather than a uniformly established alternative to antibiotics.

Phage specificity is frequently described as an ecological advantage, but specificity should not be equated with ecological predictability. Host range is influenced by receptor availability, bacterial physiological state, environmental conditions, and phage adaptation and can vary among strains (Gordillo Altamirano and Barr 2021; Venturini et al. 2022). Moreover, selective depletion of a bacterial population may indirectly influence nontarget organisms through competition, nutrient exchange, cross‐feeding, or altered metabolite production. Thus, therapeutic specificity should be evaluated at two levels: the identity of the bacterial hosts affected and the functions altered as a consequence.

Bacterial antiviral defenses further constrain phage efficacy. CRISPR‐associated (CRISPR‐Cas) systems, restriction–modification mechanisms, receptor modification or masking, abortive infection, and other defense systems can prevent productive infection and differ substantially among strains (Cui et al. 2024; Pires et al. 2020; Venturini et al. 2022). Phage selection should therefore integrate phenotypic susceptibility with genomic and physiological characterization of both phage and bacterial host, where feasible.

Phage pharmacology presents an additional translational challenge. Unlike conventional small molecules, therapeutic phages can replicate in the presence of susceptible bacteria, making exposure and pharmacodynamic effect dynamically interdependent. Absorption, distribution, tissue penetration, immune clearance, bacterial density, adsorption kinetics, and replication all contribute to therapeutic activity (Blasco et al. 2025; Kang et al. 2024; Nang et al. 2023). Standardized phage PK/PD, therefore, remains essential for rational dose selection and clinical translation.

These limitations also define the conceptual boundary of post‐microbial therapeutics. Phage therapy is an established biological approach for targeting susceptible bacteria; post‐microbial therapeutics is proposed here as an integrative framework for determining whether such targeted perturbation produces a reproducible functional, metabolic, ecological, or host‐level consequence. The latter should not be inferred from bacterial depletion alone.

Table 1 summarizes the principal advances and persistent barriers that currently determine the translational performance of phage therapy.

**Table 1:** Major advances and persistent barriers in contemporary phage therapy.

Domain	Major advances	Persistent barriers
Clinical translation	Increasing clinical investigation and compassionate‐use experience in difficult‐to‐treat bacterial infections (Petrovic Fabijan et al. 2023; Stacey et al. 2022; Strathdee et al. 2023)	Heterogeneous patients, phage preparations, treatment regimens, routes, and clinical endpoints
Host range and phage selection	Improved receptor characterization and strain‐level susceptibility testing (Gordillo Altamirano and Barr 2021; Venturini et al. 2022)	Variable host range and context‐dependent activity
Bacterial antiviral defense	Increasing genomic characterization of CRISPR‐Cas, restriction–modification, and other defense systems (Cui et al. 2024; Pires et al. 2020)	Strain‐specific resistance and reduced therapeutic predictability
Phage lifestyle and genomic safety	Genome‐based screening for undesirable lysogeny, virulence, and resistance‐associated determinants (Pires et al. 2020; Strathdee et al. 2023)	Temperate phages may promote lysogeny, gene transfer, or long‐term phenotypic change
PK/PD and biodistribution	Increasing recognition of the importance of dynamic phage exposure and bacterial response (Blasco et al. 2025; Kang et al. 2024; Nang et al. 2023)	Immune clearance, tissue barriers, bacterial density, self‐replication, and resistance complicate conventional PK/PD modeling
Manufacturing and regulation	Development of phage biobanks, purification approaches, and genomic quality‐control procedures (R. C. Lin et al. 2021; Pires et al. 2020)	Batch consistency, scalability, stability, potency, purity, and regulatory heterogeneity
Therapeutic modality	Development of whole phages, engineered phages, lysins, depolymerases, and combination products	Distinct mechanisms, pharmacology, manufacturing requirements, safety considerations, and evolutionary behavior

Abbreviation: PK/PD, pharmacokinetics/pharmacodynamics.

### 2.2 Biological Foundations Relevant to Post‐Microbial Therapeutics

Phage infection can influence bacterial systems beyond direct lysis, including gene expression, metabolism, stress responses, quorum‐associated behavior, competitive fitness, and interactions with neighboring microorganisms (Federici et al. 2021; Górski et al. 2023; Hendrix et al. 2022). These effects provide a mechanistic rationale for examining phages as selective ecological perturbation tools. However, a measurable change in bacterial physiology or community composition should not automatically be interpreted as a therapeutic benefit. Functional consequences require independent measurement and, where causal claims are made, experimental perturbation.

The distinction between virulent and temperate phages is particularly important. Virulent phages undergo productive infection followed by bacterial lysis and are generally preferred when bacterial killing is the intended mechanism. Temperate phages can establish lysogeny and persist within bacterial populations, potentially modifying bacterial phenotype, fitness, gene regulation, or susceptibility to subsequent infection. They may also contribute to horizontal gene transfer and lysogenic conversion, creating additional safety and ecological concerns (Pires et al. 2020; Strathdee et al. 2023). Therapeutic candidates should therefore undergo genomic screening for undesirable lysogeny‐associated, virulence‐related, and AMR determinants.

Bacterial physiological state is equally important. Mature biofilms can restrict adsorption and productive phage replication through nutrient limitation, spatial heterogeneity, altered receptor expression, extracellular polymeric matrices, and reduced bacterial growth. Consequently, susceptibility observed under planktonic laboratory conditions may not predict activity within chronic or device‐associated infections (J. Lin et al. 2022). Biofilm‐aware susceptibility testing is therefore necessary when the intended therapeutic target is spatially structured or physiologically heterogeneous.

Host immunity adds another layer of variability. Phage particles can interact with innate and adaptive immune mechanisms, while phage‐mediated bacterial lysis can modify local inflammatory responses (Federici et al. 2021; Górski et al. 2023). These effects are context dependent and should not be presumed to be beneficial. Similarly, phage–antibiotic interactions may be synergistic, additive, neutral, or antagonistic depending on bacterial physiology, antibiotic mechanism, dosing sequence, and phage characteristics. Metabolomic studies of phage–antibiotic combinations illustrate how functional measurements can help identify metabolic changes associated with treatment response (Han et al. 2022).

These observations provide the biological basis for the microbial‐module concept, but also impose important constraints on its use. A microbial module should not be defined simply as a group of taxa that co‐occur. Metabolic guilds, cross‐feeding consortia, and interaction‐network clusters represent related but distinct organizational concepts and should not be treated as interchangeable. Computational analyses can identify candidate modules through co‐abundance, network connectivity, pathway similarity, or metabolic complementarity; however, such associations establish hypotheses rather than functional causality. An experimentally validated functional module requires a defined biological function, evidence supporting membership and boundaries, a measurable functional output, and reproducible alteration following targeted perturbation. These distinctions are summarized in Table 2 and provide an explicit evidentiary boundary for applying the post‐microbial framework.

**Table 2:** Operational criteria for defining and validating a microbial module.

Criterion	Computationally inferred candidate module	Experimentally validated functional module
Definition	Group identified through shared abundance, network connectivity, functional annotation, or predicted metabolic relationships	Defined microbial group whose members demonstrably contribute to a specified function
Membership	Proposed from a statistical association or predicted functional similarity	Supported by mechanistic or experimental evidence of functional contribution or interaction
Function	Predicted from genomic annotation, pathway reconstruction, or metabolic modeling	Directly demonstrated using functional assays, metabolite measurements, substrate utilization, or related measurements
Co‐occurrence	Supports hypothesis generation only	Not required as evidence of functional equivalence
Module boundaries	Provisional; determined by computational clustering, network topology, or functional similarity	Defined by demonstrated functional contribution, metabolic dependence, ecological interaction, or experimentally supported necessity/sufficiency
Metabolic guild	May identify candidate members sharing resource use or metabolic potential	May contribute to a validated module when functional specialization is experimentally demonstrated; not synonymous with a module
Cross‐feeding	Predicted from complementary metabolic pathways or metabolite‐exchange models	Supported by direct evidence of metabolite transfer, reciprocal dependence, isotope tracing, supplementation/removal, or controlled co‐culture
Interaction network	Identifies putative ecological relationships	Requires experimental or mechanistic confirmation that the interaction contributes to the defined function
Functional output	Predicted	Measured reproducibly
Perturbation	In silico prediction	Addition, depletion, inhibition, genetic modification, or replacement demonstrates functional contribution
Causality	Hypothesis only	Supported when targeted perturbation reproducibly changes the defined output
Therapeutic interpretation	Candidate target for investigation	Validated functional target, subject to safety and translational evidence

### 2.3 Technological Renaissance: From Natural Phages to Engineered Platforms

Modern phage technology increasingly combines genomics, receptor biology, molecular engineering, delivery science, and quantitative screening. Whole‐genome sequencing facilitates exclusion of undesirable genetic features, whereas receptor characterization and strain‐level susceptibility testing can improve phage selection (Gordillo Altamirano and Barr 2021; Cui et al. 2024; Pires et al. 2020). Engineering approaches can further modify receptor‐binding determinants, host range, or genetic payloads.

Programmable phage systems, including CRISPR‐Cas‐based approaches, illustrate the transition from naturally occurring phages toward engineered biological platforms. Such systems may enable sequence‐specific antimicrobial or anti‐virulence functions, but engineering does not eliminate the fundamental requirements for adsorption, intracellular delivery, construct stability, and evasion of bacterial‐defense systems. Genetic programmability, therefore, expands the design space without removing the biological constraints governing phage activity.

A critical distinction must also be maintained between whole‐phage therapeutics and phage‐derived products. Whole virulent phages are replicating biological agents whose activity depends on susceptible bacterial hosts. Lysins are nonreplicating enzymes that degrade bacterial peptidoglycan, whereas depolymerases primarily target capsular or extracellular‐polysaccharide structures. Phage–enzyme combinations represent additional therapeutic modalities with distinct pharmacological, manufacturing, stability, immunogenicity, and regulatory requirements (Cui et al. 2024; Pires et al. 2020; Strathdee et al. 2023).

These differences are particularly relevant to biofilm‐associated disease. Whole phages may reduce susceptible bacterial populations, depolymerases may alter matrix or capsule architecture, and lysins may directly damage susceptible bacterial cell walls. Accordingly, evidence for one modality should not automatically be generalized to another. The therapeutic mechanism, biological endpoint, and evidence level should always be specified.

Delivery technologies, including encapsulation and local administration, may improve phage stability, tissue exposure, or protection from environmental degradation. Nevertheless, formulation cannot compensate for inappropriate host range, bacterial antiviral defenses, unfavorable biofilm physiology, or inadequate genomic safety. Effective phage development, therefore, requires simultaneous optimization of biological specificity, pharmacological exposure, ecological consequences, and product quality.

The principal mechanisms and constraints relevant to this therapeutic transition are summarized in Figure 1, which integrates direct bacterial killing with potential downstream effects on microbial functions and host responses.

**Figure 1:**
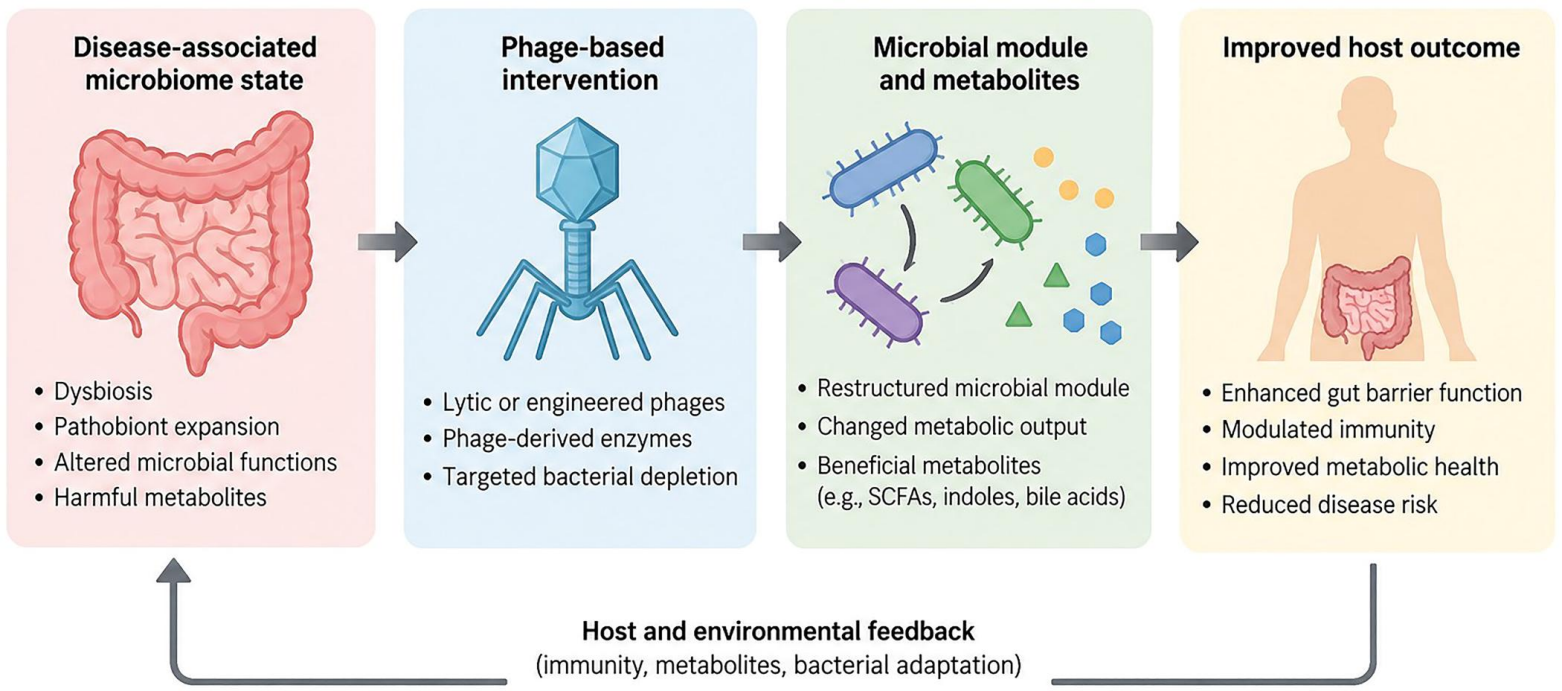
Multilayer functionality and biological constraints of therapeutic bacteriophages (designed using Canva). SCFAs, short‐chain fatty acids.

### 2.4 Lessons From Clinical Implementation

Clinical translation requires considerably more than demonstration of bacterial killing in vitro. Therapeutic performance depends on accurate phage–strain matching, genomic characterization, reproducible manufacturing, appropriate formulation, pharmacological behavior, and clinically meaningful outcome assessment. Phage biobanks may accelerate access to characterized candidates, but their value depends on standardized metadata, genomic quality control, and adequate strain coverage (R. C. Lin et al. 2021).

Phage susceptibility testing remains a major limitation because conventional assays do not fully reproduce bacterial physiology in biofilms, polymicrobial communities, or anatomically restricted niches. A strain classified as susceptible under laboratory conditions may express different receptors or antiviral defenses in vivo. Future clinical workflows should therefore integrate phenotypic susceptibility testing with genomic, physiological, and ecological information.

Patient‐level factors further influence therapeutic response. Infection site, bacterial burden, immune status, previous antimicrobial exposure, microbiome composition, and antiphage immune responses can affect phage persistence and activity (Kang et al. 2024; Nang et al. 2023). The need for improved PK/PD characterization is increasingly recognized as a major barrier to standardized clinical translation.

Regulatory evaluation must likewise distinguish among whole phages, engineered phages, lysins, depolymerases, and combination products. Differences in replication, genetic composition, pharmacology, manufacturing, evolutionary potential, and immunogenicity require product‐specific assessment of identity, purity, potency, safety, and post‐treatment monitoring (Petrovic Fabijan et al. 2023; Pires et al. 2020).

### 2.5 Toward a Function‐Centered Interpretation of Phage Therapy

Within the proposed framework, bacteriophages are best regarded as selective biological perturbation tools rather than universal substitutes for antibiotics. Their most established therapeutic role remains targeted control of bacteria, particularly when virulent phages are appropriately matched to susceptible pathogens. Their potential to alter microbial functions, metabolites, ecological interactions, or host responses represents an additional layer of investigation rather than an established therapeutic outcome (Federici et al. 2021; Rasmussen et al. 2020).

The contribution of post‐microbial therapeutics is therefore integrative rather than taxonomically novel. It does not replace phage therapy, microbiome therapeutics, ecological medicine, or other function‐based microbiome interventions. Instead, it provides a common framework for asking whether an existing or engineered microbial intervention can produce a reproducible causal sequence: target susceptibility → phage–bacterium interaction → microbial‐module perturbation → functional/metabolic response → host outcome.

This sequence also establishes an evidence hierarchy. A computationally inferred module is a hypothesis‐generating construct; a functionally characterized module has stronger mechanistic support; and an experimentally validated module requires reproducible evidence that perturbing its members changes the defined output. Co‐occurrence, network connectivity, or shared abundance alone should therefore not be interpreted as evidence of functional membership or causality.

The distinction is critical because ecosystem modulation is not inherently beneficial. Removal of a bacterial population may relieve a pathogenic function, but it may also disrupt nutrient exchange, cross‐feeding, colonization resistance, or other community functions. The relevant therapeutic question is consequently not simply whether a phage changes the microbiome, but whether a defined and validated microbial function is altered reproducibly in a direction that improves a prespecified biological or clinical outcome.

Future progress will therefore require integration of host‐range analysis, bacterial antiviral‐defense profiling, genomic safety assessment, biofilm‐aware susceptibility testing, standardized PK/PD, functional microbiome measurements, resistance surveillance, and product‐specific regulatory frameworks. This integrated approach provides a rigorous basis for extending phage therapy from selective pathogen control toward function‐directed microbial intervention while maintaining appropriate limits on current evidence.

## 3 Phage Therapy Across Disease Contexts: From Pathogen Control to Ecosystem‐Level Effects

The therapeutic rationale for bacteriophages has expanded from selective bacterial killing toward the possibility of manipulating microbial functions and ecological interactions. Nevertheless, these objectives should not be considered equivalent. Clinical and experimental evidence is strongest for phage‐mediated reduction of susceptible bacterial pathogens, whereas effects on community structure, microbial metabolism, host immunity, and disease‐associated ecological states remain more context dependent.

Within the post‐microbial framework, this distinction is important because a change in microbial abundance does not necessarily constitute a change in a validated microbial function. A phage‐induced reduction in a bacterial population may alter metabolite production, cross‐feeding, biofilm organization, or host responses, but these downstream effects require independent measurement and causal validation. Accordingly, the framework considers a sequence of phage exposure → bacterial response → microbial‐module perturbation → functional or metabolic change → host outcome, rather than assuming that ecosystem remodeling is an inherent therapeutic benefit.

### 3.1 Resistant and Difficult‐to‐Treat Bacterial Infections

The most established application of phage therapy remains the treatment of difficult‐to‐treat bacterial infections, particularly those involving multidrug‐resistant organisms for which conventional antimicrobial options are limited. Phages provide a fundamentally different mechanism from antibiotics because productive infection depends on bacterial receptor recognition, intracellular replication, and host‐cell lysis. Their narrow host range can provide substantial selectivity, potentially reducing collateral effects on nontarget bacteria. However, the same specificity creates a practical requirement for accurate pathogen characterization and appropriate phage–host matching.

This specificity is therapeutically advantageous but does not eliminate biological uncertainty. Bacterial receptor variation, physiological state, intracellular defense systems, biofilm‐associated phenotypes, and host immune clearance can all influence treatment response. Phage resistance may also emerge during treatment, sometimes through receptor modification or other bacterial adaptations. Consequently, successful therapy should be evaluated not only by phage dose but also by host range, bacterial susceptibility, PK/PD, resistance evolution, and clinically meaningful bacterial clearance.

From a post‐microbial perspective, the immediate therapeutic target in these infections is therefore usually the pathogen rather than the microbial ecosystem. Any subsequent ecological effect should be demonstrated rather than presumed. This distinction prevents the framework from conflating established antimicrobial activity with the more ambitious objective of ecosystem‐level therapeutic reprogramming.

### 3.2 Oncology and Immunocompromised Hosts

Cancer therapy can substantially perturb the intestinal microbiome through antimicrobial exposure, mucosal injury, altered nutrition, and immunosuppression. Such perturbations may reduce colonization resistance and permit expansion of opportunistic or multidrug‐resistant bacteria. Phage therapy is therefore being explored as a potentially selective means of controlling bacterial populations while avoiding some of the broad ecological effects associated with conventional antibiotics.

However, treatment in immunocompromised populations introduces additional uncertainty. Altered immune function may influence phage persistence and clearance, while mucosal injury, neutropenia, graft‐versus‐host disease, and concomitant antimicrobial treatment can modify both microbial composition and clinical outcomes. Consequently, changes in bacterial abundance or phage recovery should not be interpreted independently of inflammatory status, microbiome structure, PK/PD, and clinical endpoints.

The post‐microbial framework, therefore, requires a higher evidentiary threshold when phage therapy is proposed to produce effects beyond pathogen control. Associations among phages, bacterial taxa, metabolites, and treatment outcomes may identify candidate microbial modules or pathways, but they do not establish that a phage‐induced ecological change causes clinical benefit. A therapeutically relevant module should instead satisfy the criteria outlined in Table 3: a defined function, experimentally supported membership and boundaries, a measurable output, and reproducible alteration following targeted perturbation.

**Table 3:** Operational criteria for defining and validating a microbial module.

Criterion	Computationally inferred candidate module	Experimentally validated functional module
Primary definition	A group of microbial taxa, strains, genes, or metabolic features is identified because they show shared abundance patterns, network connectivity, functional similarity, or association with a common latent factor.	A defined microbial group whose members collectively contribute to a specified biological function and for which the functional relationship has been supported experimentally.
Functional objective	A putative function is assigned from genomic annotation, pathway reconstruction, metabolic modeling, or association with a phenotype.	The target function is explicitly defined and demonstrated through functional assays, metabolite measurements, substrate‐utilization studies, or other direct measurements.
Module membership	Membership is proposed from statistical association, network structure, shared functional annotations, or similar ecological/abundance patterns.	Membership is supported by evidence that individual members contribute to, depend on, or mechanistically influence the defined function.
Functional relationship versus co‐occurrence	Co‐occurrence or co‐abundance can support hypothesis generation, but is not sufficient evidence of functional equivalence or interaction.	Functional relationships are demonstrated independently of co‐occurrence, preferably through perturbation, complementary metabolic measurements, or defined‐community experiments.
Module boundaries	Boundaries are determined by computational criteria such as network connectivity, factorization, clustering, or functional similarity and should be considered provisional.	Boundaries are defined by functional contribution, metabolic dependency, ecological interaction, or experimentally demonstrated necessity/sufficiency.
Metabolic guild	May constitute a candidate module when taxa share a defined resource use or metabolic function.	A guild can support module membership when functional specialization or resource use is experimentally demonstrated; guild and module should not be treated as synonymous.
Cross‐feeding consortium	Candidate members may be inferred from complementary metabolic pathways or predicted exchange of metabolites.	Cross‐feeding is supported by direct evidence of metabolite transfer, reciprocal growth dependence, isotope tracing, metabolite supplementation/removal, or controlled co‐culture experiments.
Interaction‐network cluster	A cluster derived from positive/negative associations or network topology identifies putative ecological relationships.	Network structure is corroborated by experimental perturbation or mechanistic evidence showing that the inferred interaction contributes to the module‐level function.
Functional output	The expected output is predicted from pathway reconstruction, metabolic modeling, or association with a phenotype.	A measurable output—such as metabolite production, substrate conversion, resistance phenotype, biofilm phenotype, or host‐relevant response—is experimentally demonstrated.
Perturbation test	In silico perturbation can predict the consequences of removing or modifying individual members.	Targeted addition, depletion, inhibition, genetic modification, or replacement of module members demonstrates their contribution to the predicted function.
Causality	Represents a hypothesis and should not be interpreted as evidence of causality.	Causal contribution is supported when perturbation of module members produces a reproducible change in the defined functional output.
Validation	Preferably replicated in independent cohorts, data sets, environments, or analytical approaches.	Requires reproducibility across independent experiments and, where relevant, validation in defined consortia, ex vivo systems, animal models, or human studies.
Therapeutic interpretation	A candidate target for intervention or further experimental investigation.	A validated functional target that can be considered for rational microbial or phage‐mediated manipulation, subject to safety and translational evidence.

### 3.3 Musculoskeletal, Wound, and Device‐Associated Infections

Chronic osteomyelitis, diabetic wounds, burn‐associated infections, and device‐associated infections are particularly relevant to phage therapy because they frequently involve multidrug‐resistant bacteria, biofilms, impaired antimicrobial penetration, and persistent infection. Biofilm physiology can substantially alter phage–bacterium interactions through spatial heterogeneity, nutrient limitation, altered receptor expression, extracellular polymeric matrices, and changes in bacterial growth state.

These infections also illustrate why “phage‐based therapy” should not be treated as a single mechanistic category. Whole virulent phages are replicating biological agents whose activity depends on productive infection, whereas lysins are nonreplicating enzymes that hydrolyze bacterial cell‐wall components. Depolymerases can degrade extracellular polysaccharides, thereby altering bacterial accessibility within biofilms. Phage–enzyme combinations may therefore act through complementary mechanisms rather than through a single phage‐mediated process.

The ecological interpretation is similarly context dependent. Biofilm disruption may expose bacteria to antibiotics or immune mechanisms, but a reduction in biofilm biomass alone does not establish restoration of a broader microbial ecosystem. Experimental claims should therefore distinguish direct bactericidal activity, matrix degradation, altered microbial interactions, and downstream host effects. This distinction is particularly important when a treatment is described as modifying a “microbial module”: a network cluster or co‐occurring biofilm population should remain a candidate module unless its functional contribution has been experimentally demonstrated (Miao et al. 2021).

### 3.4 Gastrointestinal, Liver, and Cardiometabolic Disorders

The gastrointestinal tract provides the clearest setting in which phage therapy could extend from pathogen control toward selective manipulation of microbial functions. Gut phages can influence bacterial abundance, microbial community structure, and host–microbe interactions, while disease‐associated bacterial populations have been linked to metabolic and inflammatory phenotypes.

However, the evidence base is less mature than for direct antibacterial applications. Associations between the phageome, bacterial taxa, microbial pathways, and cardiometabolic phenotypes do not establish that phages independently drive those phenotypes. Diet, medication, host physiology, bacterial abundance, and disease state can all influence the same measurements. Thus, phage abundance or microbiome composition should not be used as a surrogate for therapeutic mechanism without functional validation.

A stronger post‐microbial strategy would begin with a defined disease‐associated microbial function. A candidate bacterial population or module could be identified computationally and linked to a measurable metabolic activity or metabolite. Phage‐mediated perturbation would then be followed longitudinally by measurement of bacterial abundance, pathway activity, metabolite production, and host phenotype. Importantly, computationally inferred modules should remain hypotheses at this stage. Co‐abundance, network connectivity, or predicted metabolic complementarity can identify candidate groups, but experimentally validated modules require evidence that their members contribute to the defined function and that targeted perturbation changes the functional output (Wu et al. 2021; Wang et al. 2023).

This distinction is central to the proposed framework. A phage that removes a bacterium associated with a disease phenotype has demonstrated selective microbial perturbation; it has not necessarily demonstrated functional ecosystem correction. The latter requires a causal chain linking bacterial perturbation to a defined microbial function, biochemical consequence, and host outcome.

### 3.5 Veterinary, Agricultural, and One Health Applications

Veterinary, agricultural, aquaculture, food, and environmental applications provide complementary evidence for the ecological dimensions of phage activity. Phages have been investigated for pathogen control across diverse biological systems, where their specificity may reduce reliance on conventional antimicrobials. These applications are relevant to a One Health perspective because antimicrobial selection and resistant organisms move across human, animal, food, and environmental compartments.

At the same time, environmental conditions can substantially alter phage performance. Temperature, pH, salinity, ultraviolet exposure, bacterial density, host physiology, community composition, and bacterial antiviral mechanisms can affect phage persistence and activity. Phage specificity also does not eliminate ecological consequences if the targeted bacterium occupies an important metabolic or community role.

These settings therefore reinforce a central principle of post‐microbial therapeutics: specificity at the bacterial level does not necessarily imply predictability at the ecosystem level. Ecological effects must be evaluated according to the function of the targeted organism within its community rather than inferred solely from taxonomic depletion. This is consistent with guild‐based approaches in microbiome research, which use functional organization to reduce dimensionality but still require experimental evidence to establish causative roles (Wu et al. 2021).

### 3.6 From Pathogen Clearance to Functional Ecosystem Modulation

Across disease contexts, the evidence supports a graded interpretation of phage action. Pathogen reduction is currently the most established therapeutic objective, particularly in difficult‐to‐treat bacterial infections. Biofilm disruption, altered bacterial physiology, and interactions with antibiotics represent mechanistically plausible extensions, but their therapeutic value is dependent on the specific phage, bacterial target, biological context, and treatment design. Broader ecosystem remodeling and correction of microbiome‐associated metabolic phenotypes remain more experimental.

This evidence gradient also clarifies how microbial modules should be incorporated into post‐microbial therapeutics. A computationally inferred module may be useful for hypothesis generation, but statistical co‐occurrence or shared abundance patterns cannot establish functional membership. Metabolic guilds, cross‐feeding consortia, and interaction‐network clusters may provide different routes for identifying candidate functional units, but they should not be treated as interchangeable. Experimental validation should demonstrate a defined function, support module membership and boundaries, measure a reproducible output, and establish that targeted perturbation changes that output (Wu et al. 2021; Wang et al. 2023).

Accordingly, the translational objective should shift from asking “Does the phage change the microbiome?” to asking “Does a defined phage perturbation reproducibly alter a validated microbial function that improves a prespecified host outcome?” This formulation preserves the established antimicrobial rationale for phage therapy while providing a more rigorous route toward ecosystem‐level applications. It also places appropriate limits on the novelty claim of post‐microbial therapeutics: the framework integrates existing phage, microbiome, ecological, and metabolic approaches around a common causal and functional sequence rather than establishing an entirely new therapeutic class.

## 4 Phage Technology and Engineering: From Natural Predators to Programmable Ecological Editors

Advances in synthetic biology, genome engineering, structural biology, materials science, and quantitative microbiology have transformed phage therapeutics from empirical isolation toward increasingly rational design. Contemporary platforms can modify receptor recognition, genomic content, payload delivery, formulation, and tissue localization, thereby extending phage applications beyond conventional bacterial killing (Gordillo Altamirano and Barr 2021; Cui et al. 2024; Pires et al. 2020). Nevertheless, the term ecological editor should be used cautiously. Engineering a phage to alter a bacterial population does not guarantee a beneficial ecosystem‐level response because outcomes depend on host range, bacterial antiviral defenses, phage lifestyle, microbial‐network structure, metabolic redundancy, host immunity, and resistance evolution. The principal engineering objective is therefore to improve the specificity, predictability, safety, and functional consequences of phage‐based intervention.

### 4.1 Genome and Receptor Engineering

Modern phage engineering employs recombineering, CRISPR‐assisted modification, synthetic genome assembly, and receptor‐binding protein redesign to improve host recognition, remove undesirable genomic elements, or introduce therapeutic payloads (Cui et al. 2024; Pires et al. 2020) (Table 4). Tail‐fiber and receptor‐binding protein engineering can broaden or refine host range, although neither maximal breadth nor extreme specificity is universally desirable. Broader infectivity may improve coverage of heterogeneous clinical isolates but may also increase ecological exposure, whereas overly restricted host range may limit therapeutic applicability (Gordillo Altamirano and Barr 2021).

**Table 4:** Major engineering strategies and translational constraints in post‐microbial phage therapeutics.

Engineering strategy	Principal objective	Evidence maturity	Major translational constraints
Genome and receptor engineering	Refine host recognition, genomic safety, and payload delivery	Preclinical to early translational	Genetic stability, host‐range trade‐offs, bacterial defenses, and resistance evolution
Programmable/CRISPR phages	Sequence‐specific targeting of resistance or virulence functions	Predominantly preclinical	Intracellular delivery, CRISPR/RM barriers, construct stability, and ecological effects
Whole virulent phages	Replicating target‐dependent bacterial killing	Clinical use and ongoing investigation	Host‐range variability, immune clearance, resistance, and complex PK/PD
Lysins	Direct peptidoglycan degradation	Preclinical to translational	Delivery, spectrum, stability, and immunogenicity
Depolymerases	Capsule or extracellular‐polysaccharide degradation	Predominantly preclinical	Substrate specificity and biofilm penetration
Phage–enzyme combinations	Complement bacterial killing with matrix disruption	Early translational	Product complexity, potency definition, compatibility, and regulation
Delivery systems	Improve stability, localization, and controlled release	Predominantly preclinical	Manufacturing reproducibility, carrier safety, and maintenance of phage viability
Metabolic/ecological steering	Modify defined microbial functions or metabolite outputs	Early experimental stage	Causal validation, network compensation, and long‐term ecological safety

Abbreviation: PK/PD, pharmacokinetics/pharmacodynamics.

Programmable phages can also deliver sequence‐specific antimicrobials or anti‐virulence constructs. However, successful activity still requires adsorption, intracellular delivery, payload expression, and escape from bacterial‐defense mechanisms. Genome engineering can additionally improve biosafety by excluding undesirable genetic determinants. This is particularly important when distinguishing virulent from temperate phages: virulent phages are generally preferred for direct antibacterial therapy, whereas temperate phages raise concerns related to lysogeny, bacterial phenotype modification, persistence, and horizontal gene transfer (Pires et al. 2020; Strathdee et al. 2023).

### 4.2 Engineering Around Bacterial Antiviral Defenses

Bacterial antiviral defenses are major determinants of phage infectivity and should be considered explicitly during engineering. CRISPR‐Cas systems, restriction–modification pathways, receptor alteration or masking, abortive‐infection mechanisms, and other intracellular defenses can generate marked differences in susceptibility even among strains of the same species (Cui et al. 2024; Venturini et al. 2022) (Table 4).

Engineering strategies may partially overcome these barriers by modifying receptor‐binding proteins, targeting alternative receptors, or combining phages with complementary infection routes. However, such interventions may also impose new selective pressures and promote bacterial escape. Phage development should therefore integrate target‐strain genomics, phenotypic susceptibility testing, antiviral‐defense profiling, resistance‐evolution studies, and longitudinal monitoring rather than relying on taxonomic matching alone.

### 4.3 Phage‐Derived Enzymes and Biofilm‐Directed Engineering

Whole phages and phage‐derived enzymes are mechanistically distinct therapeutic modalities. Whole virulent phages are replicating biological agents whose activity depends on adsorption, bacterial physiology, intracellular replication, and completion of the lytic cycle. In contrast, lysins are nonreplicating enzymes that cleave bacterial peptidoglycan, whereas depolymerases primarily degrade capsular or extracellular polysaccharides. Their pharmacology, manufacturing, evolutionary behavior, and regulatory requirements therefore differ substantially (Cui et al. 2024; Pires et al. 2020).

These distinctions are especially important in biofilm‐associated infection. Biofilms contain extracellular matrices, nutrient and oxygen gradients, slow‐growing cells, and heterogeneous receptor expression that can reduce phage accessibility and replication (Bhuiyan 2025; J. Lin et al. 2022). Depolymerases may improve matrix penetration, whereas lysins can provide direct cell‐wall‐targeting activity under appropriate conditions. Phage–enzyme combinations may offer complementary mechanisms, but synergy should be demonstrated experimentally rather than assumed. Accordingly, biofilm‐directed engineering should evaluate both structural disruption and bacterial physiological heterogeneity.

### 4.4 Delivery and Formulation Technologies

Phages face physiological barriers, including gastric acidity, mucus, immune clearance, tissue compartmentalization, and limited residence time at infection sites. Encapsulation in hydrogels, polymeric carriers, lipid‐based systems, or other biomaterials may improve stability, localization, and controlled release. Such approaches are particularly relevant for gastrointestinal, wound, respiratory, and other anatomically restricted applications.

Delivery technologies, however, cannot compensate for unsuitable host range, strong bacterial antiviral defenses, unfavorable biofilm physiology, or unsafe genomic content. Formulation must therefore be integrated with phage biology. In post‐microbial applications, spatially restricted delivery may also reduce unnecessary perturbation of microbial communities outside the target niche, although this possibility requires rigorous biodistribution and ecological‐safety assessment.

### 4.5 Cell‐Free and High‐Throughput Design Platforms

Cell‐free transcription–translation systems, automated host‐range mapping, single‐cell assays, and high‐throughput genomic screening are accelerating phage prototyping and candidate selection. These platforms allow rapid comparison of engineered variants using quantitative parameters, such as adsorption efficiency, killing kinetics, host range, resistance emergence, and genetic stability.

Their principal value lies in design and screening, not in reproducing the complexity of infection in vivo. Biofilm architecture, immune interactions, community composition, metabolite availability, and anatomical barriers remain difficult to model in simplified systems. Engineered candidates, therefore, require validation across increasingly complex biological models before clinical translation.

### 4.6 Phage–Metabolite Interactions as Engineering Targets

Phage infection can directly influence bacterial metabolism and indirectly alter the extracellular metabolic environment through cell lysis, competition, and community restructuring. Experimental studies have demonstrated metabolic reprogramming during defined phage–bacterium interactions and phage–antibiotic combinations (Han et al. 2022; Hendrix et al. 2022). These findings justify the incorporation of metabolomics into phage‐engineering studies when functional outcomes extend beyond bacterial killing.

However, engineered phages should not yet be described as established metabolic actuators. Metabolite changes following phage exposure may arise from direct bacterial responses, release of intracellular contents, or secondary ecological restructuring, and their downstream effects depend on community composition and host physiology. A mechanistically rigorous strategy should therefore proceed from disease‐relevant microbial function → responsible microbial module → targeted phage perturbation → measured metabolic change → validated host response. Integration with engineered nutritional or metabolite‐producing platforms may eventually support combined functional interventions, but such approaches remain developmental (Bhuiyan 2026). The principal engineering strategies are summarized in Figure 2.

**Figure 2:**
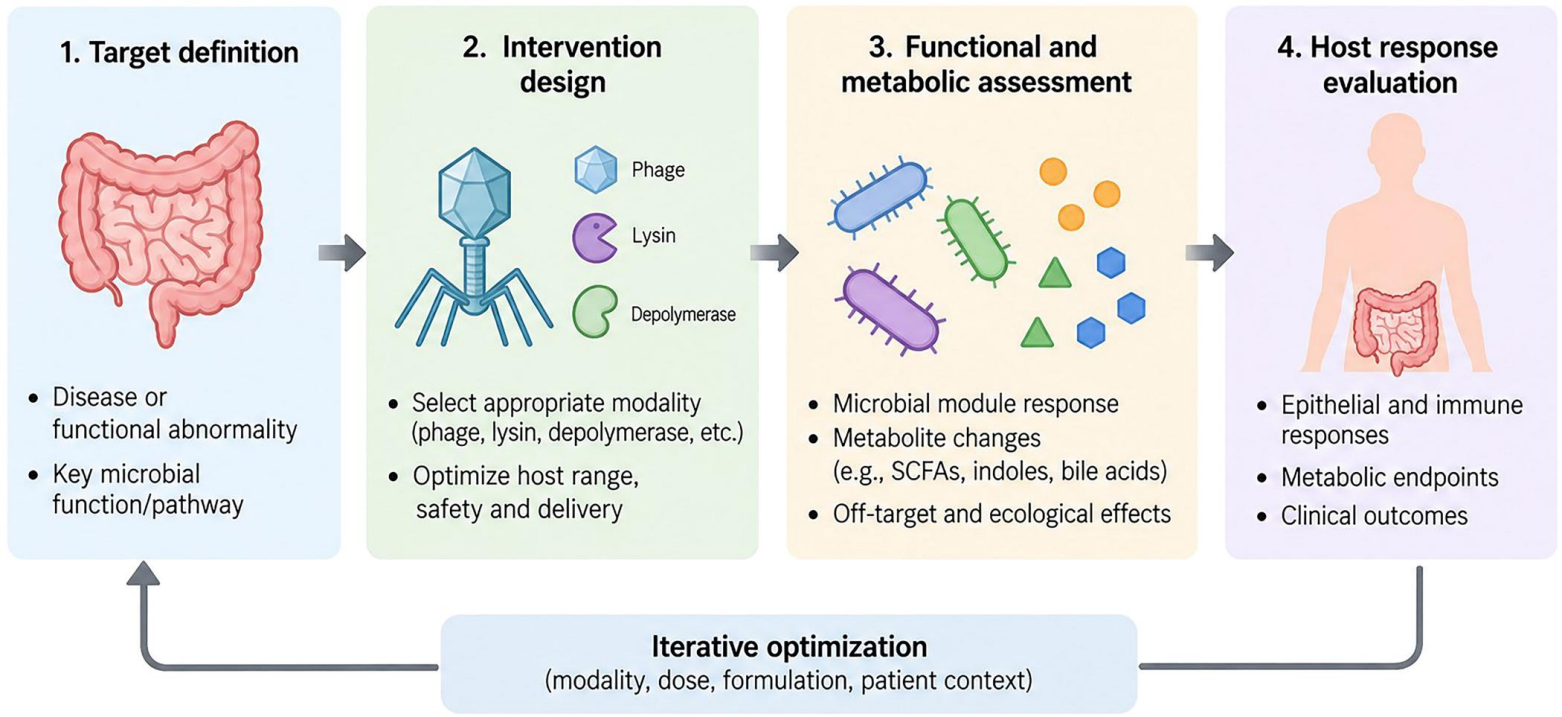
Translational framework for post‐microbial therapeutics (designed using Canva). SCFAs, short‐chain fatty acids.

Conceptual overview of four major engineering domains: genome and receptor engineering for host recognition, genomic safety, and programmable payload delivery; phage‐derived lysins and depolymerases as distinct nonreplicating modalities; formulation and delivery technologies that improve stability and localization; and metabolic or ecological steering through defined microbial perturbation (Table 4). Therapeutic performance is constrained by bacterial antiviral defenses, phage lifestyle, biofilm physiology, immune clearance, resistance evolution, and microbial‐network structure. Downstream metabolic and host effects should therefore be interpreted as context dependent and experimentally validated rather than assumed consequences of phage administration (Gordillo Altamirano and Barr 2021; Cui et al. 2024; Hendrix et al. 2022; Pires et al. 2020; Venturini et al. 2022).

### 4.7 Safety, Evolution, and Regulatory Considerations

Engineering increases both therapeutic flexibility and biosafety requirements. Phage and bacterial populations can evolve during treatment, generating receptor changes, altered defense systems, resistance, or changes in bacterial fitness. Engineered phages raise additional concerns regarding genetic stability, recombination, environmental persistence, and unintended transfer of engineered sequences.

Phage lifestyle remains a central safety consideration. Therapeutic development generally favors genomically characterized virulent phages, whereas temperate phages require greater caution because of lysogeny and potential genetic exchange. Candidate phages should therefore be screened for lysogeny‐associated genes, known virulence determinants, and antimicrobial‐resistance genes before clinical development (Pires et al. 2020; Strathdee et al. 2023).

Regulatory requirements should also reflect therapeutic modality. Whole virulent phages, engineered phages, lysins, depolymerases, nanocarrier formulations, and phage–enzyme combinations differ in replication capacity, composition, pharmacology, manufacturing, and evolutionary potential. A single regulatory model is therefore unlikely to be appropriate across all phage‐derived products. Product‐specific assessment of identity, potency, genomic safety, stability, manufacturing consistency, and post‐treatment surveillance will be required (Petrovic Fabijan et al. 2023).

### 4.8 Translational Implications: Toward Functional Precision

The future of phage engineering will not be determined solely by increasing genomic complexity. Translational success will depend on whether engineered interventions produce reproducible antibacterial effects and, where relevant, measurable ecological or metabolic outcomes without unacceptable evolutionary or immunological risks.

For infectious diseases, the primary requirement remains effective and safe pathogen control. For microbiome‐directed applications, a higher evidentiary threshold is necessary: the intervention should be linked to a defined microbial function, a measurable metabolic consequence, and a clinically relevant host response. Phage development should therefore integrate genomic safety, lifestyle assessment, receptor biology, bacterial‐defense profiling, host‐range testing, biofilm activity, PK/PD, biodistribution, resistance evolution, and functional endpoints.

Overall, engineering has created the technical capacity to move phages from naturally occurring antibacterial agents toward increasingly programmable therapeutic platforms. However, their designation as ecological editors is justified only when targeted perturbation can be linked experimentally to predictable functional outcomes. Achieving this goal will require the integration of genomic engineering, bacterial antiviral defense profiling, phage lifestyle selection, biofilm‐aware testing, quantitative pharmacology, functional metabolomics, and evolution‐aware safety assessment. Such integration provides the technological foundation for precision post‐microbial therapeutics while directly addressing the biological and translational limitations.

## 5 Therapeutic Role of Microbial Metabolites, Metabolic Flux, and Nutrition Interfaces

Microbial metabolites provide a functional interface between microbial communities and host physiology. Unlike taxonomic profiles, which describe community composition, metabolite patterns reflect the combined biochemical activities of microorganisms, dietary substrates, host metabolism, and local environmental conditions. Short‐chain fatty acids (SCFAs), indole derivatives, secondary bile acids, polyamines, and related metabolites can influence epithelial integrity, immune regulation, inflammatory signaling, and systemic metabolism (Malogan et al. 2025; Zeng et al. 2025). Within the post‐microbial framework, these metabolites are therefore considered potential mechanistic intermediates linking microbial perturbation to host response rather than merely downstream biomarkers.

However, metabolite associations should not be equated automatically with therapeutic causality. A clinically relevant metabolite should ideally be linked to an identifiable microbial pathway or module, show a reproducible change after a defined intervention, and contribute experimentally to a measurable host phenotype. This distinction is essential because metabolite concentrations are shaped simultaneously by microbial production, host metabolism, diet, absorption, and ecological interactions (Federici et al. 2021; Malogan et al. 2025).

### 5.1 Microbial Metabolites as Functional Mediators

SCFAs are among the best‐characterized microbiota‐derived metabolites. Acetate, propionate, and butyrate participate in epithelial, immunological, and metabolic regulation, with butyrate particularly associated with the maintenance of intestinal barrier function and immune homeostasis (Zeng et al. 2025). Tryptophan‐derived indoles can activate host receptors, such as the aryl hydrocarbon receptor, thereby influencing mucosal immunity and epithelial responses, whereas microbially transformed bile acids participate in metabolic and inflammatory signaling.

These observations support functional analysis of microbial metabolism but do not establish metabolomics as universally superior to taxonomy. Taxonomic and metabolic measurements are complementary: microbial profiling identifies candidate producers or consumers, whereas metabolomics reveals biochemical outputs. The strongest mechanistic inference, therefore, arises from the integration of both layers.

Phage intervention can alter metabolite profiles through several mechanisms. Productive infection may reprogram bacterial metabolism before lysis, while bacterial depletion can modify substrate competition, cross‐feeding, and community pathway activity. Lysis may also release intracellular nutrients into the surrounding environment (Hendrix et al. 2022). These mechanisms should be distinguished because immediate lysis‐associated release differs from secondary ecosystem‐level metabolic restructuring.

Targeted phage activity may modify bacterial abundance, metabolic pathways, substrate competition, and cross‐feeding, thereby altering pools of SCFAs, indole derivatives, bile‐acid metabolites, and other bioactive compounds. These changes may influence epithelial, immune, and metabolic pathways, but their magnitude and direction depend on microbial‐module structure, phage host range, bacterial antiviral defenses, diet, and host physiology. Metabolites are therefore represented as potential mechanistic intermediates requiring causal validation rather than universal mediators of phage‐associated benefit (Gan et al. 2023; Hendrix et al. 2022; Malogan et al. 2025; Zeng et al. 2025).

### 5.2 Bidirectional Phage–Metabolite Interactions

Phage–metabolite interactions are bidirectional. Bacterial metabolic state, nutrient availability, receptor expression, and intracellular physiology can influence susceptibility to phage infection, while phage activity can modify the chemical environment of microbial communities. Phage‐mediated lysis may release amino acids, nucleotides, lipids, and other substrates that become available to neighboring organisms, potentially altering competitive and cooperative interactions.

Experimental studies support this functional relationship. Phage‐associated modulation of *Pseudomonas aeruginosa* has been linked to altered bacterial metabolic behavior, while phage–antibiotic synergy against multidrug‐resistant *K. pneumoniae* has been associated with distinct changes in metabolic pathways (Han et al. 2022; Hendrix et al. 2022). Similarly, selective phage targeting of metabolically abnormal *K. pneumoniae* was associated with improvement in nonalcoholic fatty liver disease‐related phenotypes (Gan et al. 2023).

These findings support incorporating metabolomics into mechanistic phage studies, but they do not justify describing phage therapy as inherently “metabolic therapy.” A more defensible interpretation is that phage intervention can generate metabolic consequences, some of which may contribute to therapeutic outcome when supported by temporal and causal evidence.

### 5.3 Nutrition and Synthetic‐Biology Interfaces

Diet shapes the metabolic environment in which phage–bacterium interactions occur. Fermentable carbohydrates, amino acids, polyphenols, lipids, and micronutrients influence bacterial physiology, community composition, and metabolite production. Consequently, the ecological effect of targeting the same bacterium may differ across nutritional environments, particularly when alternative organisms can compensate for the disrupted function.

Nutritional manipulation may eventually complement phage therapy by modifying substrate availability or supporting restoration of beneficial metabolic activity. However, dietary interventions affect multiple microbial and host pathways simultaneously and therefore require independent mechanistic validation.

Synthetic biology offers an additional interface through engineered microbial or yeast‐based platforms capable of producing defined bioactive compounds. Yeast‐derived pharmafood concepts illustrate how controlled biochemical outputs might complement microbiome‐directed intervention (Bhuiyan 2026). Such combinations remain developmental and should not be presented as established clinical phage therapies. Their value will depend on whether they correct a defined functional deficit rather than simply adding nutritional or metabolic supplementation.

### 5.4 Microbial Modules and Metabolic Niches

Within this framework, a microbial module is defined as a functionally coherent set of microorganisms and/or microbial pathways that collectively perform a measurable ecological or biochemical process. A module may correspond to a metabolic guild, cross‐feeding consortium, interaction‐network cluster, or taxonomically diverse group sharing a defined functional output.

This operational definition directly addresses the limitation of taxonomy‐centered analysis. Multiple organisms may perform the same metabolic function, whereas a single organism may participate in several modules depending on the environmental context. Consequently, depletion of one taxon may produce little functional change when redundancy is high, but disruption of a nonredundant or highly connected organism may alter an entire pathway.

Modules can be inferred computationally by integrating metagenomic, transcriptomic, metabolomic, and ecological‐network data and then validated experimentally using defined communities, targeted perturbations, pathway‐specific assays, or metabolite‐rescue approaches. Their therapeutic relevance, therefore, lies in experimentally demonstrated function rather than statistical co‐occurrence alone.

### 5.5 Major Metabolite Classes Relevant to Post‐Microbial Therapy

Microbial metabolites differ substantially in their biological functions, sources, and evidentiary links to phage intervention. Some classes, including SCFAs, indole derivatives, and secondary bile acids, have well‐established roles in epithelial, immune, and metabolic regulation, whereas direct evidence that therapeutic phages reproducibly alter these metabolites remains limited. Phage‐associated metabolic effects may arise through selective depletion of metabolite‐producing bacteria, altered cross‐feeding, changes in microbial pathway activity, or release of intracellular substrates after lysis. These mechanisms are highly context dependent and are influenced by diet, microbial redundancy, host metabolism, and community structure. Accordingly, metabolite measurements should be interpreted as functional readouts of microbial perturbation rather than assumed mediators of therapeutic benefit. The major metabolite classes relevant to post‐microbial therapy are summarized in Table 5.

**Table 5:** Major metabolite classes relevant to post‐microbial therapy.

Metabolite class	Major host‐related function	Potential relationship with phage perturbation	Key limitation
Short‐chain fatty acids (SCFAs)	Regulation of epithelial integrity, mucosal immunity, and host metabolism	Phage‐mediated changes in bacterial fermentation networks may alter SCFA production	Strong dependence on diet, microbial redundancy, intestinal absorption, and host metabolism
Indole derivatives	AhR‐associated epithelial and immune signaling	Perturbation of tryptophan‐metabolizing populations may modify indole‐related pathways	Multiple microbial taxa contribute to the same metabolic pathways
Secondary bile acids	Regulation of metabolism, inflammation, and colonization resistance	Selective alteration of bile‐acid‐transforming bacteria may modify bile‐acid pools	Bile‐acid profiles reflect coordinated microbial and host metabolism
Amino acids and related intermediates	Microbial cross‐feeding, host metabolism, and cellular signaling	Phage‐mediated lysis may redistribute intracellular nutrients to neighboring organisms	Nutrient release alone does not establish a beneficial therapeutic effect
Polyamines	Cellular growth, epithelial physiology, and microbial‐community regulation	Changes in producer or consumer populations may alter local polyamine concentrations	Direct evidence linking therapeutic phages to clinically relevant polyamine changes remains limited
Lipid‐ and energy‐related metabolites	Energy metabolism and inflammatory signaling	Phage‐driven ecological restructuring may indirectly modify lipid‐ and energy‐associated pathways	Causal attribution is difficult because of strong dietary and host metabolic influences

Abbreviation: AhR, aryl hydrocarbon receptor.

The strength of evidence linking phage intervention to these metabolite classes remains uneven. SCFAs, indoles, and bile acids have well‐characterized physiological roles, but direct evidence that therapeutic phages reproducibly modify these pathways and thereby improve clinical outcomes remains limited (Gan et al. 2023; Kirk et al. 2024; Malogan et al. 2025; Zeng et al. 2025). Future studies should therefore combine targeted metabolomics with defined phage perturbation, microbial‐module analysis, and host‐response measurements to distinguish causal metabolic effects from secondary ecological associations.

### 5.6 Extending Metabolite‐Focused Phage Research Beyond Infection

Phage–metabolite relationships are increasingly investigated in cardiometabolic and other noninfectious conditions. Associations have been reported among gut phage communities, bacterial metabolic pathways, circulating metabolites, insulin sensitivity, and lipid‐related phenotypes (Kirk et al. 2024; Wortelboer and Herrema 2024). However, such observations do not establish therapeutic causality because changes in the phageome may reflect alterations in bacterial hosts, diet, medication exposure, or disease state.

The same caution applies to immunocompromised and transplantation settings, where phage–bacterium configurations may correlate with metabolic or clinical outcomes. Translation requires intervention studies demonstrating that selective phage perturbation modifies a defined microbial function and produces a clinically relevant host response. In these contexts, the post‐microbial framework is most useful when the therapeutic target is a measurable dysfunctional pathway rather than bacterial abundance alone.

### 5.7 Measurement, Causality, and Standardization

A major barrier to metabolite‐centered phage therapy is the lack of standardized approaches linking phage exposure to metabolic function. Untargeted metabolomics can identify broad biochemical changes but often provides limited attribution to specific organisms or pathways. Variation in sampling, analytical platforms, annotation, and normalization further complicates comparison across studies (Malogan et al. 2025).

More rigorous causal analysis should combine longitudinal sampling, targeted metabolomics, defined microbial communities, and, where appropriate, stable‐isotope tracing or metabolic‐network modeling. Computationally predicted pathways and modules should be regarded as hypotheses until experimentally validated. The key mechanistic sequence is: phage exposure → target‐bacterial response → microbial‐module change → metabolic alteration → host‐functional response.

Clinical studies should therefore integrate phage exposure, target‐bacterial abundance, resistance emergence, relevant metabolites, and host outcomes rather than relying on isolated omic measurements.

### 5.8 Conceptual Integration

Current evidence supports positioning microbial metabolites as measurable functional intermediates within post‐microbial therapeutics rather than as universally beneficial therapeutic endpoints. A rational workflow should begin with a disease‐relevant functional abnormality, identify the associated metabolite or pathway, map the responsible microbial module, assess phage susceptibility and bacterial antiviral defenses, apply a targeted perturbation, and determine whether the resulting metabolic change improves host function.

This framework can be summarized as functional abnormality → metabolite/pathway identification → microbial‐module mapping → phage susceptibility and defense profiling → targeted intervention → metabolic‐flux measurement → host‐response validation.

The principal value of metabolomics is therefore not simply to add another analytical layer, but to connect microbial perturbation with experimentally testable biochemical and host outcomes. When integrated with functional module definition, phage biology, and causal validation, this approach provides the biochemical foundation for the phage–module–metabolite model developed in the following section.

## 6 Integrative Model Linking Phages, Microbial Modules, Metabolites, and Host Function

Phages, microbial communities, metabolites, and host responses form an interconnected biological system rather than independent therapeutic domains. Selective phage pressure can alter susceptible bacterial populations, with downstream effects on microbial interactions, metabolic pathways, and host physiology. Conversely, bacterial state, nutrient availability, metabolites, host immunity, and antiviral defense systems can modify phage infectivity and persistence (Federici et al. 2021; Hendrix et al. 2022; Rasmussen et al. 2020; Venturini et al. 2022). The post‐microbial framework, therefore, treats therapy as a sequence of measurable perturbations across interacting biological layers.

The model is organized around three operational components: phages as selective perturbation tools, microbial modules as functionally coherent units, and metabolites as measurable biochemical intermediates. These relationships are conditional rather than deterministic. A phage alters a module only when the targeted bacterium is both susceptible and functionally relevant; compositional changes may have little metabolic consequence when functional redundancy is high; and metabolite changes are therapeutically meaningful only when causally linked to host outcomes.

### 6.1 Architecture of the Phage–Module–Metabolite Triad

The proposed phage–module–metabolite–host axis links selective microbial perturbation to functional and host‐level consequences (Figure 3). At the first layer, phages impose selective pressure on susceptible bacteria, with the magnitude and duration of the effect determined by receptor availability, bacterial physiology, local exposure, antiviral defense, spatial organization, and phage lifestyle (Pires et al. 2020; Strathdee et al. 2023; Venturini et al. 2022). Virulent phages primarily mediate productive infection and lysis, whereas temperate phages can establish lysogeny and influence bacterial fitness, gene exchange, and longer‐term community dynamics. These lifestyles, therefore, represent distinct therapeutic and ecological processes.

**Figure 3:**
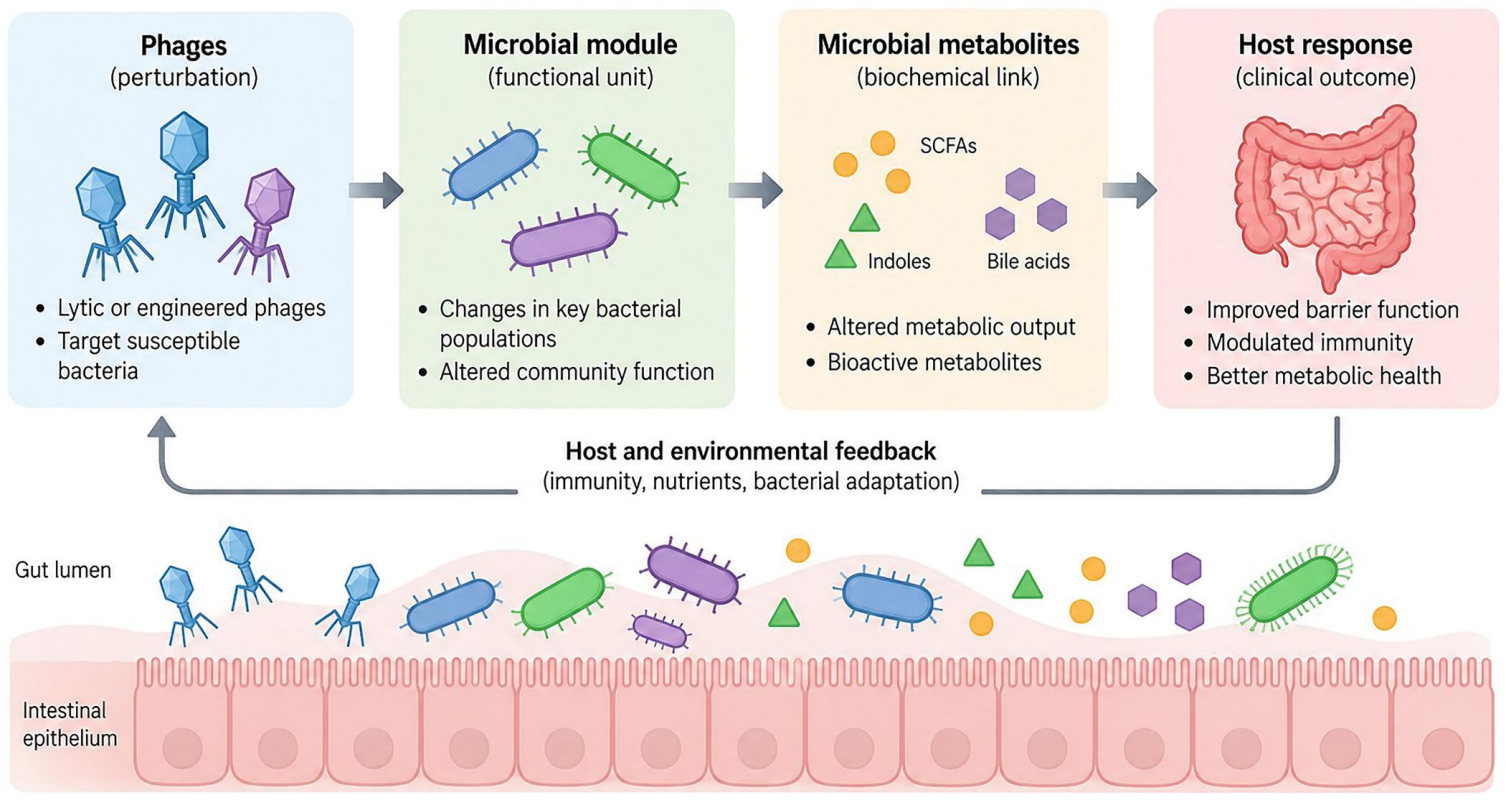
Gastrointestinal phage–microbial module–metabolite–host axis (designed using Canva). SCFAs, short‐chain fatty acids.

The second layer comprises microbial modules, defined here as functionally bounded microbial units that contribute to a specified biological process and generate a measurable output. Metabolic guilds, cross‐feeding consortia, and network clusters may serve as starting points for module identification, but they are not equivalent to validated functional modules without evidence of contribution or dependence. Multiomics and network analyses can nominate candidate modules, whereas defined‐community reconstruction, targeted perturbation, pathway‐specific measurements, isotope tracing, or metabolite‐rescue experiments can establish functional membership and boundaries (Khan Mirzaei and Deng 2022; Rasmussen et al. 2020).

The third layer comprises microbial metabolites, which provide measurable biochemical links between microbial activity and host physiology. Changes in bacterial abundance, pathway activity, substrate availability, or cross‐feeding can alter SCFAs, indole derivatives, bile‐acid metabolites, and other bioactive compounds, affecting epithelial, immune, and metabolic pathways (Malogan et al. 2025; Zeng et al. 2025). Thus, the axis is not strictly linear: bacterial physiology and metabolites can influence phage activity, while host immunity can affect phage persistence and microbial composition (Federici et al. 2021; Górski et al. 2023).

Figure 3 summarizes this bidirectional architecture: phage perturbation acts on a defined bacterial target; the resulting ecological change can alter the activity of a functional module; module‐level changes can modify metabolic flux and metabolite availability; and these biochemical changes may influence epithelial, immune, or systemic host functions. Conversely, host immunity, bacterial resistance, spatial structure, and metabolite availability can modify phage infectivity and persistence. The figure, therefore, represents a testable causal architecture rather than an assumed biological sequence. Importantly, a change in bacterial abundance or metabolite concentration alone does not demonstrate module‐level mediation or therapeutic benefit.

### 6.2 From Association to Mechanistic Causality

A central challenge in microbiome therapeutics is distinguishing association from causation. Parallel changes in the phageome, bacteriome, metabolome, and host phenotype may reveal biologically informative relationships but cannot establish their direction or mediation. The framework therefore prioritizes a perturbation‐first strategy in which a defined intervention is followed longitudinally across microbial, metabolic, and host levels (Figure 3).

A rigorous experimental sequence is defined phage exposure → target‐bacterial response → functional‐module change → metabolic‐flux alteration → host‐functional response (Table 6). Each transition should be measured independently using appropriate controls. Candidate modules identified computationally should be tested through targeted depletion, resistant bacterial controls, defined‐community reconstruction, add‐back experiments, isotope tracing, or metabolite supplementation or depletion. Such experiments can determine whether the proposed module is functionally required and whether its metabolic output mediates the downstream phenotype.

**Table 6:** Major knowledge gaps linking phage, microbial‐module, metabolite, and host layers.

Knowledge gap	Principal layers	Core problem	Priority strategy
Variable phage efficacy	Phage–bacterium–module	Host‐range variation, receptor heterogeneity, antiviral defense, and resistance reduce predictability	Combine receptor mapping, susceptibility testing, defense profiling, and longitudinal resistance monitoring
Uncertain functional effects	Phage–module	Targeted taxa may be redundant or functionally central	Integrate targeted perturbation with network and functional validation
Association rather than causality	Module–metabolite–host	Parallel multiomic changes do not establish a mechanism	Use longitudinal perturbation, defined communities, and metabolite‐rescue studies
Dynamic PK/PD	Phage–bacterium–host	Replication, immune clearance, and biodistribution alter exposure	Develop quantitative phage PK/PD linked to susceptible‐host density
Lifestyle‐associated safety	Phage–bacterium–community	Temperate phages may establish lysogeny or facilitate genetic exchange	Prefer genomically characterized virulent phages for conventional lytic therapy
Biofilm‐associated failure	Phage–module–microenvironment	Matrix barriers and slow growth reduce accessibility and replication	Apply biofilm‐specific susceptibility testing and modality‐specific combinations
Unvalidated ecological dosing	All layers	No validated metric links phage exposure to ecosystem function and clinical benefit	Prospectively integrate PK/PD with module, metabolite, resistance, and host endpoints
Regulatory heterogeneity	Product–host	Whole phages, engineered phages, lysins, depolymerases, and combinations have different risk profiles	Develop modality‐specific quality, safety, and monitoring standards

Abbreviation: PK/PD, pharmacokinetics/pharmacodynamics.

The study by Gan et al. (2023), in which selective phage targeting of high‐alcohol‐producing *K. pneumoniae* was associated with improvement in metabolic and hepatic phenotypes, illustrates the value of linking a defined microbial target with a measurable host outcome. However, it does not by itself establish that a specific microbial module or metabolite mediates the observed effect. Accordingly, Figure 3 should be interpreted as a framework for testing causal links, not as evidence that every connection has already been experimentally established.

### 6.3 Ecological Dose and Phage PK/PD

Conventional PK/PD remains essential for phage therapy, but living phages differ from static drugs because their replication depends on susceptible bacterial density and their persistence is influenced by immune clearance, anatomical distribution, and local environmental conditions (Blasco et al. 2025; Kang et al. 2024; Nang et al. 2023) (Table 6).

Within this framework, ecological dose is proposed only as a conceptual extension of conventional exposure–response analysis. It refers to the magnitude and duration of biological perturbation required to produce a defined functional change in a microbial module without unacceptable ecological or host effects. It is not currently a validated clinical dosing parameter.

A future ecological‐dose model could integrate phage exposure, bacterial susceptibility, receptor availability, antiviral defenses, resistance emergence, module restructuring, metabolite trajectories, and host responses. Importantly, this concept cannot be applied uniformly across whole phages, lysins, depolymerases, engineered phages, and combination products because these modalities differ fundamentally in replication and pharmacology. Prospective validation is required before ecological dose can inform clinical decision‐making.

### 6.4 Translational Decision Framework

Clinical implementation should integrate taxonomy with functional measurements rather than replacing one with the other. Taxonomic and genomic data remain essential for identifying bacterial targets and selecting phages, while metabolomics and pathway analysis can reveal functional consequences of intervention.

A practical workflow begins by defining the clinical or functional abnormality, followed by identifying the responsible microbial module. Phage selection should then incorporate host‐range testing, receptor compatibility, and bacterial antiviral‐defense profiling, including CRISPR‐Cas and restriction–modification systems (Gordillo Altamirano and Barr 2021; Venturini et al. 2022). The appropriate therapeutic modality should next be selected whole virulent phage, engineered phage, lysin, depolymerase, or combination product according to the biological target and clinical context (Pires et al. 2020; Strathdee et al. 2023).

Functional response should then be measured longitudinally through bacterial abundance, phage kinetics, resistance emergence, module structure, metabolite trajectories, and relevant host endpoints. Adjunctive interventions, including antibiotics, nutrition, or metabolite support, should be added only when a complementary mechanism is experimentally justified. Han et al. (2022), for example, showed metabolic reorganization associated with phage–antibiotic synergy, but such findings do not establish the general superiority of multimodal therapy.

### 6.5 Biofilm and Quorum‐Regulated Behavior

Biofilm‐associated infection illustrates the importance of integrating structural, physiological, and ecological factors (Table 6). Mature biofilms contain extracellular polymeric matrices, nutrient gradients, slow‐growing cells, altered receptor expression, and metabolically heterogeneous populations that can reduce phage replication and antibiotic susceptibility (Bhuiyan 2025; J. Lin et al. 2022).

Quorum sensing contributes to matrix production, virulence, and community organization, but its therapeutic manipulation remains species‐ and context‐dependent. Whole virulent phages, lysins, and depolymerases should therefore be distinguished mechanistically: intact phages require productive infection, lysins directly target bacterial cell walls, and depolymerases degrade specific capsules or extracellular polysaccharides. Biofilm intervention can consequently be considered at three levels: structural disruption, bacterial population control, and restoration of local function.

### 6.6 Computational and Systems Tools

Computational methods can help integrate phage genotype, receptor recognition, bacterial‐defense systems, microbial‐network structure, metabolite production, host immunity, and PK/PD. Network inference can identify candidate microbial modules, pathway reconstruction can link genes to metabolic outputs, and longitudinal multiomic integration can distinguish coordinated from sequential responses (Blasco et al. 2025; Nang et al. 2023).

Machine learning and digital‐twin concepts may eventually support host‐range prediction, module identification, resistance forecasting, and individualized intervention design. However, these approaches remain investigational and should not be considered clinically validated decision tools. Predictions must be tested against experimental susceptibility, biological outcomes, and standardized data sets because receptor expression, bacterial antiviral defenses, and physiological state can substantially alter phage behavior.

### 6.7 Integrative Perspective

The value of the phage–module–metabolite model lies in converting ecosystem‐centered therapy into a set of testable biological relationships. Phages are selective perturbation agents rather than intrinsically beneficial ecological editors; microbial modules are experimentally defined functional units rather than simple co‐occurrence clusters; and metabolites are measurable intermediates whose causal relevance must be demonstrated. A rigorous post‐microbial strategy can therefore be summarized as clinical functional abnormality → microbial‐module identification → phage susceptibility and antiviral‐defense profiling → modality‐specific intervention → ecological and metabolic measurement → host‐function validation → clinical outcome assessment.

This model extends beyond taxonomy‐centered microbiome manipulation by explicitly linking intervention, microbial function, biochemical response, and host outcome while preserving the distinction between established phage therapy and more experimental ecosystem‐engineering applications.

## 7 Cross‐Cutting Challenges, Ethics, and Regulatory Pathways

Translation of post‐microbial therapeutics requires regulatory and ethical frameworks capable of accommodating biologically distinct interventions, including whole phages, engineered phages, lysins, depolymerases, metabolites, and combination products (Table 7). These modalities differ in replication capacity, pharmacology, evolutionary behavior, manufacturing requirements, and ecological risk and therefore should not be regulated as a single therapeutic class. Evaluation should retain conventional standards of quality, safety, and efficacy while incorporating bacterial antiviral defenses, resistance evolution, genetic exchange, microbiome effects, and functional host outcomes where relevant (Cui et al. 2024; Petrovic Fabijan et al. 2023; Pires et al. 2020).

**Table 7:** Governance priorities for post‐microbial therapeutics.

Domain	Priority requirement	Rationale
Evolutionary safety	Resistance and bacterial‐defense surveillance	CRISPR‐Cas, restriction–modification, receptor changes, and other defenses can alter phage efficacy
Genomic safety	Distinguish virulent from temperate phages	Lysogeny and horizontal gene transfer create additional risks
Ecological safety	Assess effects beyond the direct host range	Specific targeting may indirectly perturb keystone functions and microbial networks
Quality control	Standardize identity, purity, stability, and potency	Reproducibility is essential for clinical translation
Product regulation	Distinguish whole phages, engineered phages, lysins, depolymerases, and combinations	These modalities differ in replication, pharmacology, manufacturing, and evolutionary behavior
Functional endpoints	Validate ecological and metabolomic biomarkers	Multiomics signals require causal and analytical validation
Ethics and equity	Transparent consent and scalable access	Personalized ecosystem‐directed therapies may increase uncertainty and inequity

Within the post‐microbial framework, ecological and metabolomic measurements should complement rather than replace microbiological and clinical endpoints. Regulatory assessment should therefore be product‐specific, mechanism‐aware, and function‐informed, with evidentiary requirements proportional to the complexity and intended biological effect of each intervention.

### 7.1 Safety in an Evolutionary and Ecological Landscape

Phage therapy operates within an evolutionary system. Bacterial resistance may emerge through receptor modification, CRISPR‐Cas immunity, restriction–modification systems, abortive‐infection mechanisms, and other antiviral defenses, thereby altering therapeutic efficacy during treatment (Cohan et al. 2020; Cui et al. 2024; Venturini et al. 2022). Resistance surveillance and bacterial‐defense profiling should therefore be incorporated into safety assessment and longitudinal monitoring.

Phage lifestyle is equally important. Virulent phages are generally preferred for conventional antibacterial therapy, whereas temperate phages may establish lysogeny and influence bacterial phenotype, fitness, horizontal gene transfer, and long‐term community behavior (Pires et al. 2020; Strathdee et al. 2023). Therapeutic candidates should therefore undergo whole‐genome screening for lysogeny‐associated genes, known virulence factors, antimicrobial‐resistance determinants, and other undesirable genetic features.

Ecological safety cannot be inferred from a narrow host range alone. Even a highly specific phage may indirectly alter nontarget organisms if it removes a keystone or metabolically important bacterial population. Cross‐feeding, nutrient competition, and metabolite production may consequently change despite limited direct killing of commensal taxa (Gordillo Altamirano and Barr 2021; Rasmussen et al. 2020; Wahida et al. 2021).

Safety assessment must also distinguish among therapeutic modalities. Whole phages are replicating biological entities with evolutionary potential, whereas lysins and depolymerases are nonreplicating biological macromolecules. Engineered phages introduce additional concerns regarding genetic stability and recombination, while combination products require evaluation of interactions among active components. Metabolite‐directed interventions similarly require monitoring of systemic effects because microbial metabolites can influence immune, epithelial, endocrine, and metabolic pathways (Malogan et al. 2025; Zeng et al. 2025).

### 7.2 Standardization, Quality Control, and Potency

Lack of harmonized analytical and manufacturing standards remains a major obstacle to reproducible phage therapy. Clinical products require reliable characterization of identity, genomic content, host range, purity, potency, stability, and susceptibility profile. Phage biobanks can facilitate rapid therapeutic matching, but their value depends on standardized metadata, bacterial strain characterization, genomic quality control, and reproducible storage and release procedures (R. C. Lin et al. 2021).

Plaque assays remain useful but do not fully predict activity within biofilms, polymicrobial communities, or anatomically restricted infection sites. Receptor expression, bacterial physiological state, antiviral defenses, immune clearance, and local microenvironmental conditions may substantially alter in vivo activity (Nang et al. 2023; Venturini et al. 2022). Potency assessment should therefore be modality specific. Whole phages require infectivity and killing measurements; lysins require enzymatic activity assays; depolymerases require substrate‐specific degradation assays; and engineered or combination products require additional confirmation of genetic stability and component compatibility.

For post‐microbial applications, validated ecological or metabolomic endpoints may eventually complement conventional potency measurements. However, multiomics complexity alone does not establish regulatory relevance. Functional biomarkers must demonstrate analytical reproducibility, mechanistic validity, and a meaningful relationship to therapeutic outcome.

### 7.3 Product‐Specific and Function‐Informed Regulation

The diversity of phage‐based interventions challenges conventional regulatory models. A naturally occurring virulent phage, an engineered phage, a purified lysin, and a personalized phage cocktail have fundamentally different biological characteristics and should not be evaluated under identical requirements (Petrovic Fabijan et al. 2023; Pires et al. 2020).

A defensible regulatory approach is therefore product‐specific but function‐informed. Whole phages should be evaluated for infectivity, genomic safety, host range, resistance evolution, and dynamic PK/PD. Engineered phages require additional assessment of genetic stability, payload behavior, and off‐target effects. Lysins and depolymerases should be assessed as distinct nonreplicating biological products, with attention to substrate specificity, stability, and immunogenicity. Combination products require independent characterization of each active component and their interactions.

Compassionate‐use programs have been important for patients with otherwise difficult‐to‐treat infections, but compassionate use should not be interpreted as proof of clinical efficacy. Routine implementation will require prospective trials, standardized clinical endpoints, reproducible manufacturing, and structured post‐treatment surveillance (Petrovic Fabijan et al. 2023; Strathdee et al. 2023).

### 7.4 Ethics, Equity, and Ecological Responsibility

Ethical evaluation should reflect the uncertainty associated with living and ecosystem‐directed therapies. Informed consent should distinguish established antibacterial objectives from exploratory claims involving microbiome remodeling or metabolic restoration. This distinction is particularly important for engineered phages and interventions intended for chronic, noninfectious disease, where the evidentiary base remains limited (Anomaly 2020).

Equitable access represents another challenge. Personalized phage matching, genome sequencing, metabolomics, and advanced susceptibility testing may concentrate treatment within highly specialized centers. Standardized phage libraries, scalable manufacturing, rapid diagnostics, and simplified clinical workflows will therefore be important for preventing precision phage therapy from widening existing health disparities.

Data governance should also be considered because microbiome‐directed therapy may depend on genomic, metagenomic, metabolomic, and clinical data sets. Transparent data‐use policies and appropriate consent are necessary. For replicating or engineered phages, environmental persistence, shedding, and interaction with bacterial populations outside the intended therapeutic niche may also warrant monitoring.

### 7.5 Clinical Implementation

Clinical implementation depends on rapid pathogen identification, access to characterized phages, timely susceptibility testing, reproducible product preparation, and integration with established antimicrobial and surgical management. Phage therapy should therefore develop alongside antimicrobial stewardship rather than as a competing therapeutic system.

Microbiome‐directed applications will require additional infrastructure, as clinical laboratories currently focus primarily on pathogen detection rather than on functional microbial profiling. Validated assays will be needed to distinguish clinically meaningful functional abnormalities from normal inter‐individual variation. Training across infectious disease, clinical microbiology, pharmacology, bioinformatics, and regulatory science will consequently be essential.

Overall, governance of post‐microbial therapeutics should align regulatory requirements with the mechanism, product class, evolutionary potential, and intended functional effect of the intervention, rather than treating all phage‐derived technologies as equivalent.

## 8 Future Directions and Research Priorities

The future of post‐microbial therapeutics depends on converting a function‐centered concept into experimentally validated and clinically reproducible interventions. Technological complexity alone will not establish therapeutic value. A successful strategy must demonstrate that a defined intervention modifies an identifiable microbial function, produces a measurable biochemical consequence, and improves a clinically relevant host outcome without unacceptable evolutionary or ecological effects.

### 8.1 Programmable and Hybrid Therapeutics

Engineered phages may increasingly enable the modification of host recognition, the delivery of sequence‐specific antimicrobial payloads, and the manipulation of defined bacterial functions. However, these platforms remain constrained by adsorption specificity, bacterial antiviral defenses, genetic stability, resistance evolution, and biosafety (Cui et al. 2024; Pires et al. 2020).

The concept of Living Medicines 2.0 is therefore best regarded as a forward‐looking research framework rather than an established therapeutic category. Future systems may combine selective phage perturbation with engineered microbial producers, metabolite delivery, or nutritional support. Yeast‐derived pharmafood platforms illustrate one potential route for controlled production of bioactive compounds, but their integration with phage therapy requires direct evidence of functional benefit (Bhuiyan 2026).

Future combination strategies should therefore follow a mechanistic principle: identify the functional abnormality, define the microbial module responsible, introduce a selective perturbation, and add biochemical support only when experimentally justified.

### 8.2 Computational Models and Digital Twins

Computational modeling may help integrate phage genotype, bacterial susceptibility, antiviral‐defense architecture, microbial‐network structure, metabolite production, host immunity, and PK/PD. Machine‐learning approaches could assist with host‐range prediction, resistance forecasting, microbial‐module identification, and candidate prioritization.

Patient‐specific digital twins may eventually simulate alternative microbiome interventions before treatment. However, clinically validated digital‐twin systems for phage therapy are not yet available. Their development will require longitudinal patient data sets, standardized multiomic measurements, experimentally validated network relationships, and prospective testing against observed outcomes (Khan Mirzaei and Deng 2022). Computational models should therefore support hypothesis generation and therapeutic prioritization rather than replace phenotypic susceptibility testing.

### 8.3 Expansion Beyond Infectious Disease

The strongest clinical rationale for phage therapy remains targeted control of bacterial infection. Extension into cardiometabolic, inflammatory, or other microbiome‐associated diseases requires stronger causal evidence. Associations between the gut phageome, insulin sensitivity, lipid metabolism, and related phenotypes suggest biological relevance but do not establish therapeutic efficacy (Kirk et al. 2024; Wortelboer and Herrema 2024).

Future studies should begin with a defined disease‐related microbial function rather than an altered phageome signature. A candidate microbial module should be linked mechanistically to a relevant metabolite or pathway, followed by selective perturbation and longitudinal assessment of microbial, metabolic, and host responses. Gan et al. (2023) illustrate this type of mechanistic approach.

Risk tolerance should also vary by indication. Greater uncertainty may be acceptable in an otherwise untreatable multidrug‐resistant infection than in the elective manipulation of microbiome function in chronic disease.

### 8.4 Establishing Causal Phage–Module–Metabolite Pathways

The central scientific priority is to establish causal relationships connecting phage intervention with host benefit. Parallel changes in phage, bacterial, metabolite, and host profiles are insufficient to demonstrate the mechanism. Future studies should test the sequence: defined phage exposure → target‐bacterial response → microbial‐module restructuring → metabolic change → host‐functional outcome.

Causal inference can be strengthened through resistant bacterial controls, defined microbial communities, metabolite‐rescue experiments, longitudinal sampling, targeted metabolomics, and appropriate host models. This requirement is particularly important because the novelty of post‐microbial therapeutics depends on linking microbial intervention to function rather than merely describing ecosystem associations.

### 8.5 Toward Validated Ecological PK/PD

Conventional phage PK/PD should remain the foundation of clinical dosing. Phage concentration, susceptible bacterial density, replication kinetics, biodistribution, immune clearance, and the emergence of resistance remain essential determinants of therapeutic response (Blasco et al. 2025; Kang et al. 2024; Nang et al. 2023).

The proposed ecological dose may eventually extend this framework by incorporating microbial‐module restructuring, metabolite changes, and host‐functional responses. However, it remains a conceptual research construct. Prospective studies must first establish reproducible relationships among phage exposure, bacterial susceptibility, antiviral‐defense profiles, ecological changes, metabolic responses, and clinical outcomes before ecological dosing can guide patient‐specific treatment.

### 8.6 Phage and Functional‐Data Infrastructure

Future phage repositories should extend beyond sequence and host‐range information. Standardized annotation of bacterial receptors, antiviral defenses, resistance pathways, biofilm activity, formulation compatibility, PK/PD, and functional outcomes could substantially improve therapeutic matching and reproducibility (R. C. Lin et al. 2021).

A future phage–function repository could connect characterized phages with bacterial targets, genomic safety data, susceptibility profiles, documented resistance mechanisms, and experimentally validated ecological or metabolic effects. Functional annotations should clearly distinguish reproducible direct effects from context‐dependent observations because metabolite responses vary with diet, microbiome composition, anatomical location, and host physiology.

### 8.7 Equity and Responsible Innovation

Responsible innovation requires that scalability and access be considered during technology development. Personalized phage cocktails, sequencing, metabolomics, and computational modeling may otherwise remain restricted to highly specialized centers. Standardized libraries, scalable production, rapid diagnostics, and simplified decision frameworks should therefore accompany increasingly sophisticated technologies.

Terminology should also reflect evidentiary maturity. Concepts such as ecological editing, metabolic steering, digital twins, and Living Medicines 2.0 are useful research directions but should not imply a degree of clinical control that has not yet been demonstrated.

### 8.8 Priority Research Agenda

Translation of post‐microbial therapeutics will require coordinated progress across molecular phage biology, microbial ecology, metabolomics, pharmacology, and regulatory science. The most urgent priorities are those that reduce uncertainty between phage exposure, microbial perturbation, functional change, and host outcome. In particular, causal validation, bacterial‐defense profiling, phage‐lifestyle assessment, and modality‐specific comparison are necessary to improve therapeutic predictability and safety. Equally important are standardized approaches for validating microbial modules, measuring metabolite responses, and relating conventional phage PK/PD to broader ecological effects.

These priorities also reflect the current limitations of the field. Many proposed ecosystem‐level effects remain based on association rather than intervention‐level evidence, while clinically relevant differences among virulent and temperate phages, whole‐phage products, lysins, depolymerases, and engineered platforms require clearer evaluation. Long‐term ecological surveillance and harmonized regulatory standards will therefore be essential before function‐centered phage interventions can be implemented reproducibly across clinical settings. The principal research priorities and their translational objectives are summarized in Table 8.

**Table 8:** Priority research agenda for post‐microbial therapeutics.

Priority	Key research need	Translational objective
Causal validation	Link defined phage perturbation to microbial function, metabolite change, and host outcome	Establish a therapeutic mechanism
Bacterial‐defense profiling	Characterize CRISPR‐Cas, restriction–modification, receptor changes, and other defenses	Improve phage selection and predictability
Phage‐lifestyle safety	Compare virulent and temperate phages across genomic and ecological outcomes	Define appropriate therapeutic phage classes
Modality comparison	Compare whole phages, lysins, depolymerases, engineered phages, and combinations	Match modality to disease context
Functional‐module validation	Experimentally validate computationally inferred microbial modules	Convert network associations into therapeutic targets
Metabolomic standardization	Establish reproducible, mechanistically relevant metabolite endpoints	Develop functional biomarkers
Ecological PK/PD	Relate phage exposure to ecological and host‐functional effects	Validate ecological‐dose concepts
Long‐term ecological safety	Monitor effects on nontarget microbial functions	Define acceptable ecological risk
Regulation and implementation	Develop product‐specific quality, safety, and clinical standards	Enable harmonized and equitable translation

Abbreviation: PK/PD, pharmacokinetics/pharmacodynamics.

Collectively, these priorities emphasize that the field should progress from descriptive multiomics toward mechanistically validated and clinically interpretable intervention studies. A successful research agenda should therefore integrate molecular phage characterization, bacterial antiviral‐defense profiling, functional microbial ecology, metabolite measurement, longitudinal PK/PD, and host‐response assessment within the same experimental framework. This approach would provide the evidence required to determine which elements of post‐microbial therapeutics are ready for clinical translation and which remain exploratory.

### 8.9 Future Perspective

The long‐term value of post‐microbial therapeutics will depend on whether they provide a more predictive and experimentally useful framework than taxonomy‐centered microbiome manipulation. Its principal contribution is the explicit linkage of phage susceptibility, microbial‐module function, metabolite dynamics, and host response within a testable therapeutic sequence.

Future interventions are therefore more likely to involve precisely characterized biological tools directed at defined microbial functions than indiscriminate ecosystem engineering. Virulent‐versus‐temperate phage selection, bacterial antiviral‐defense profiling, modality‐specific regulation, causal metabolomics, quantitative PK/PD, and long‐term functional monitoring will be central to this transition.

If these requirements are met, phage therapeutics may progress from empirically matched antibacterial preparations toward mechanism‐guided biological interventions. Until then, programmable ecological editing, digital twins, metabolic steering, and Living Medicines 2.0 should remain clearly presented as research priorities rather than established clinical capabilities. This distinction maintains scientific rigor while providing a realistic roadmap for the development of post‐microbial precision medicine.

## 9 Conclusion

Post‐microbial therapeutics should be understood as a testable integrative framework, not as a newly established therapeutic class. Its practical contribution is to connect a defined microbial perturbation with a validated microbial function, a measurable biochemical consequence, and a clinically relevant host outcome. The framework is strongest where each step can be experimentally separated and tested. Phage therapy provides the clearest model for this logic because bacterial susceptibility can be measured directly, but whole phages, engineered phages, lysins, depolymerases, delivery systems, and combinations have distinct mechanisms and evidence requirements. A microbial module should likewise not be equated with co‐occurrence: computationally inferred modules are hypotheses until perturbation, reconstruction, flux, or rescue experiments establish functional contribution. Current evidence supports selective bacterial control and several mechanistic effects of phage, whereas claims of durable ecosystem restoration or metabolite‐mediated clinical benefit remain less mature. The next phase of the field should therefore prioritize causal validation, standardized phage PK/PD, function‐specific biomarkers, resistance and ecological surveillance, and product‐specific regulation. The value of the framework will ultimately be determined not by the novelty of its terminology, but by whether it improves the prediction, testing, and reproducibility of microbiome‐directed interventions.

## Author Contributions

**Mohammad Nazrul Islam Bhuiyan:** conceptualization, writing – review and editing, visualization, project administration, supervision, resources, writing – original draft. **Maliha Momtaj:** writing – original draft.

## Funding

The authors have nothing to report.

## Ethics Statement

The authors have nothing to report.

## Conflicts of Interest

The authors declare no conflicts of interest.

## Declaration of Generative AI and AI‐Assisted Technologies in the Writing Process

ChatGPT was employed solely to improve the clarity, grammar, and overall readability of the review paper. These tools were not used for content generation or idea development, and all original content was conceived and written by the author.

## Data Availability

The authors have nothing to report.
